# *Trypanosoma cruzi* specific mRNA amplification by in vitro transcription improves parasite transcriptomics in host-parasite RNA mixtures

**DOI:** 10.1186/s12864-017-4163-y

**Published:** 2017-10-16

**Authors:** Rafael Luis Kessler, Daniela Parada Pavoni, Marco Aurelio Krieger, Christian Macagnan Probst

**Affiliations:** 1 Functional Genomics Laboratory, Instituto Carlos Chagas, FIOCRUZ, Curitiba, PR Brazil; 2 Bioinformatics and Computational Biology Laboratory, Instituto Carlos Chagas, FIOCRUZ, Curitiba, PR Brazil

**Keywords:** *Trypanosoma cruzi*, Trypanosomatid, RNA-Seq, Transcriptomics, RNA amplification, T7 RNA polymerase

## Abstract

**Background:**

Trypanosomatids are a group of protozoan parasites that includes the etiologic agents of important human illnesses as Chagas disease, sleeping sickness and leishmaniasis. These parasites have a significant distinction from other eukaryotes concerning mRNA structure, since all mature mRNAs have an identical species-specific sequence of 39 nucleotides at the 5′ extremity, named spliced leader (SL). Considering this peculiar aspect of trypanosomatid mRNA, the aim of the present work was to develop a *Trypanosoma cruzi* specific in vitro transcription (IVT) linear mRNA amplification method in order to improve parasite transcriptomics analyses.

**Methods:**

We designed an oligonucleotide complementary to the last 21 bases of *T. cruzi* SL sequence, bearing an upstream T7 promoter (T7SL primer), which was used to direct the synthesis of second-strand cDNA. Original mRNA was then amplified by IVT using T7 RNA polymerase. T7SL-amplified RNA from two distinct *T. cruzi* stages (epimastigotes and trypomastigotes) were deep sequenced in SOLiD platform. Usual poly(A) + RNA and and T7-oligo(dT) amplified RNA (Eberwine method) were also sequenced. RNA-Seq reads were aligned to our new and improved *T. cruzi* Dm28c genome assembly (PacBio technology) and resulting transcriptome pattern from these three RNA preparation methods were compared, mainly concerning the conservation of mRNA transcritional levels and DEGs detection between epimastigotes and trypomastigotes.

**Results:**

T7SL IVT method detected more potential differentially expressed genes in comparison to either poly(A) + RNA or T7dT IVT, and was also able to produce reliable quantifications of the parasite transcriptome down to 3 ng of total RNA. Furthermore, amplification of parasite mRNA in HeLa/epimastigote RNA mixtures showed that T7SL IVT generates transcriptome quantification with similar detection of differentially expressed genes when parasite RNA mass was only 0.1% of the total mixture (R = 0.78 when compared to poly(A) + RNA).

**Conclusions:**

The T7SL IVT amplification method presented here allows the detection of more potential parasite differentially expressed genes (in comparison to poly(A) + RNA) in host-parasite mixtures or samples with low amount of RNA. This method is especially useful for trypanosomatid transcriptomics because it produces less bias than PCR-based mRNA amplification. Additionally, by simply changing the complementary region of the T7SL primer, the present method can be applied to any trypanosomatid species.

**Electronic supplementary material:**

The online version of this article (10.1186/s12864-017-4163-y) contains supplementary material, which is available to authorized users.

## Background

Transcriptomics is a relatively new area of life sciences that aims to analyze the complete set of expressed RNAs in a specific cell or organism, under specific conditions, using high-throughput techniques [1]. Initially based on DNA microarrays [2], nowadays transcriptomics is mainly performed by RNA-Seq [3]. This method consists in deep sequencing of cDNA, followed by transcript identification and quantification based on the alignment of millions of sequences (reads) to a reference genome [4], resulting in a quantitative digital map of RNA expression [5]. RNA-Seq is a very powerful technique [1, 6], but one limitation is the initial RNA mass required for entering the pipeline (generally 100 ng for poly(A) + RNA). To circumvent input limitations, RNA amplification is an interesting option, and recent developments have enabled transcriptome analysis from few or even single cells [7, 8].

Three main RNA amplification methods are mostly used: (i) in vitro transcription (IVT), (ii) PCR-based and (iii) rolling circle amplification (reviewed by [8]). IVT protocol was initially developed in the Eberwine laboratory [9], and is based on reverse transcription with an oligo(dT) primer bearing an upstream T7 promoter, which directs in vitro transcription with T7 RNA polymerase (amplification) after synthesis and purification of double-strand cDNA. The linear amplified material provides a more precise estimation of transcript levels in comparison to PCR-based exponential amplification [10]. As microarrays required a higher RNA mass when compared to RNA-Seq, IVT was commonly used to produce enough material for array hybridization [11], and no significant bias was introduced by RNA amplification [12–14]. Besides, a more reproducible expression profile from a wide range of RNA inputs is produced, improving the reliability of array results regardless of sample expansion [12, 13]. Hence, IVT mRNA amplification was extensively used for preparing RNA samples for array analysis [15, 16] and it was recently adapted for RNA-Seq [17] and single-cell RNA-Seq [18–20].

Trypanosomatids are a group of human pathogenic parasites that includes the etiological agents of Chagas disease (*Trypanosoma cruzi*), sleeping sickness (*Trypanosoma brucei*) and leishmaniasis (*Leishmania* sp) [21]. Trypanosomatids have a significant distinction from other eukaryotes concerning RNA transcription and processing: all mature mRNAs are produced after the processing of polycistronic precursors by concerted trans-splicing and polyadenylation reactions [22]. Through trans-splicing, all mature mRNA have the same 39 nucleotides species-specific sequence at the 5′ region, called mini-exon or spliced leader (SL) [23]. This characteristic allows the use of the SL sequence for specific analyzes of all mRNA from trypanosomatids. The “spliced leader trapping” method [24] enriches for 5´-SL extremity of mRNA by using a SL specific primer for second-strand cDNA synthesis and to further amplify 5´-SL containing fragments by PCR, enabling simultaneous mapping of 5’splice sites and profiling of corresponding gene expression on RNA-Seq experiments. Similar protocols have been used to map splice sites [25–27] and to measure changes in splice site usage during *T. brucei* life cycle [24, 26]. Detailed protocols to generate RNA-Seq libraries enriched for 5′-SL mRNA extremity were recently published [28, 29] and a web server platform for quantitative identification of SL and polyadenylation sites in kinetoplastid genomes is also available [30]. Recently, using a method similar to SL trapping, Mulindwa and collaborators [31] used SL primers to synthesize *T. brucei*-specific cDNA on host-parasite RNA mixtures, followed by PCR amplification using nested primers to amplify parasite mRNA. Although introducing significant bias in comparison to poly(A) + RNA, the authors considered that PCR based amplification method of SL-containing mRNAs allowed comparison of different samples as long as they were all treated equally [31].

Here, we describe the use of *T. cruzi* SL sequence for parasite-specific mRNA amplification. We demonstrate a *T. cruzi* specific IVT amplification method based in second-strand cDNA synthesis using a SL primer bearing an upstream T7 promoter (T7SL). RNA-Seq analyses of two distinct forms of *T. cruzi* (epimastigotes and trypomastigotes) show that T7SL amplification does not introduce significant bias in RNA-Seq quantification and also allows parasite transcriptome analysis in mixed RNA samples. This method presents better performance, measured by Pearson correlation, than the recently published PCR-based *T. brucei* mRNA amplification [31], enabling better transcriptome quantification, especially for samples with low amount of RNA or host-parasite mixtures.

## Methods

### Cell culture

Parasite culture: *T. cruzi* Dm28c epimastigotes [32] were cultured at 28 °C in LITB medium supplemented with 10% heat-inactivated fetal bovine serum (FBS) as previously described [33]. Tissue culture-derived trypomastigote forms were obtained by infection of cultured Vero cell (ATCC® CCL-81™) at 37 °C in a humidified 5% CO_2_ atmosphere using a multiplicity of infection (MOI) of 10 parasites per host cell. Trypomastigotes were recovered after 4 days of infection, in the cell burst peak.

### Fluorescence activated cell sorting (FACS)

Flow cytometry and cell sorting experiments were performed in a FACSAriaII (Becton-Dickinson, San Jose, CA, USA) using a 85 μm nozzle and 45 psi sheet pressure. A total of 10,000 events were acquired in the regions previously identified as corresponding to *T. cruzi* cells [34]. For epimastigote cell sorting, a FSC-A vs SSC-A gate comprising about 90% of all singlets were used. One hundred thousand (10^5^) epimastigotes were sorted directly to 700 μl of RNA extraction buffer (buffer RLT of Qiagen RNeasy kit) kept under 4 °C during sorting. Total sorting volume was ~200 μl. To the resulting cell lysate (900 μl), 250 μl of 100% ethanol was added and RNA was purified by RNA clean-up protocol of Qiagen handbook with additional on-column DNase digestion step.

### RNA purification

Total RNA was extracted from *T. cruzi* epimastigote and trypomastigote forms (5 × 10^8^ cells) with the RNeasy kit (Qiagen, Hilden, Germany), according to the manufacturer’s instructions, with an additional on-column DNase digestion step. Total RNA from HeLa cells were obtained from MessageAmp™ II aRNA Amplification Kit from Life Technologies^®^ (#AMB1751-5). RNA integrity was assessed on an Agilent 2100 Bioanalyzer with RNA 6000 Nano LabChip kit, according to the manufacturer’s instructions.

Polyadenylated RNA were purified from at least 50 μg of total RNA using PolyA + Track^®^ mRNA Isolation System III from Promega^®^ (#Z5300), according to the manufacturer’s instructions.

### RNA amplification

All mRNA amplification reactions were done using reagents from MessageAmp™ II aRNA Amplification Kit from Life Technologies^®^ (#AMB1751-5). For classic Eberwine amplification method, first-strand cDNA was synthesized using a T7oligo(dT) primer that contains a T7 RNA polymerase promoter upstream to the poly-T tract; this promoter will further direct the in vitro mRNA synthesis (amplification). First, 100 ng of total RNA (in 11 μl) were mixed with first-strand cDNA reaction mix containing 1 μl of T7oligo(dT) primer, 2 μl first-strand buffer, 4 μl dNTPs, 1 μl RNase inhibitor and 1 μl ArrayScript Reverse Transcriptase and incubated for 2 h at 42 °C. Second-strand cDNA was then synthesized for 2 h at 16 °C using the total 20 μl first-strand product plus 80 μl of second strand mix containing 63 μl water, 10 μl second-strand buffer, 4 μl dNTPs, 2 μl DNA polymerase and 1 μl of RNase H. Double-strand cDNA was then purified using PureLink^®^ PCR Micro Kit from Life Technologies (#K310250) according to manufacturer’s instructions. Eluted cDNA was adjusted to 16 μl and used as template for amplification reaction in a total volume of 40 μl containing 16 μl NTPs, 4 μl amplification buffer and 4 μl of T7 RNA polymerase; in vitro transcription took 14 h at 37 °C. Amplified RNA (aRNA) was then purified using RNeasy MinElute CleanUp Kit (Qiagen, #74204), according to manufacturer’s instructions.

For specific amplification of spliced leader containing mRNA, we used a custom designed primer complementary to the last 21 bases of *T. cruzi* spliced leader (in bold) with an upstream T7 RNA polymerase promoter (in lower case) (5′-ggccagtgaattgtaatacgactcactatagggaGGCGG**TACAGTTTCTGTACTATATTG**-3′), which we named T7SL. In this case, T7SL was used for second-strand cDNA synthesis, while first-strand cDNA was produced using random primers, according to the second round amplification protocol of MessageAmp™ II aRNA Kit. Template total RNA was adjusted to 10 μl, mixed with 2 μl of “second round primers” (random primers) and incubated at 70 °C for 10 min followed by snap cooling on ice to allow annealing of random primers to the RNA. Then, 8 μl of the reverse transcription master mix was added and first-strand cDNA was synthesized at 42 °C for 2 h. RNase H was added (1 μl) and the sample incubated at 37 °C for 30 min to specifically degrade the remaining RNA. T7SL primer was added to a final concentration of 1 μM and the sample was incubated at 70 °C for 10 min and then placed on ice. Second-strand cDNA master mix without RNase H was added (74 μl) and the reaction incubated for 2 h at 16 °C. Double-strand cDNA purification, in vitro transcription and aRNA purification was performed as described above.

For a more stringent second-strand cDNA synthesis directed by T7SL primer, we also developed a modified protocol using Platinum^®^ Pfx DNA Polymerase (Life Technologies, #11708013) for second-strand cDNA synthesis at 68 °C instead of 16 °C, and primer annealing at a higher temperature, when using the DNA polymerase of MessageAmp™ II aRNA Kit. In this case, after RNA degradation with RNase H, single-strand cDNA were mixed with T7SL primer (1 μM) and Platinum^®^ Pfx DNA polymerase mix (5 μl 10× Pfx amplification buffer, 1 μl 50 mM MgSO_4_ and 1 μl polymerase) in a final volume of 50 μl. Sample was placed on a thermal cycler as following: 95 °C for 3 min (denaturing), 55 °C for 10 min (T7SL annealing) and 68 °C for 30 min (second-strand cDNA synthesis). cDNA purification, in vitro transcription and aRNA purification was performed as described above.

### RNA quantification and length distribution assessment

Amplified RNA was quantified using Qubit RNA HS Assay Kit (#Q32852) in a Qubit^®^ 2.0 fluorometer (Life Technologies). For length distribution analysis, aRNA concentration was adjusted to 1 ng μl^−1^ and analyzed on an Agilent 2100 Bioanalyzer with RNA 6000 Pico LabChip kit, according to the manufacturer’s instructions.

### RNA-Seq

#### Sample preparation and sequencing

Negative control aRNA samples, generated from pure HeLa RNA, were sequenced in Omega Bio-Tek Inc. (Norcross, GA, USA) using the Illumina 2 × 100 paired-end sequencing method. All other samples were sequenced in the DNA Sequencing platform of Instituto Carlos Chagas (Curitiba, PR, Brazil) using SOLiD™ 1 × 50 single-end sequencing method.

For SOLiD™ sequencing, Total RNA-Seq Kit (Life Technologies, #4445374) was used accordingly to manufacturer’s instructions. Briefly, RNA was fragmented using RNase III and cleaned up with Invitrogen RiboMinus™ Concentration Module. Strand-specific adaptors were hybridized and ligated to both extremities of RNA in an overnight reaction, followed by first-strand cDNA synthesis with reverse transcriptase. cDNA was purified using MinElute® PCR Purification Kit (Qiagen) and gel-based selected for size using a Novex TBE-Urea 6% gel (#EC6865bOX). On-gel cDNA was amplified by PCR using SOLiD™ RNA barcoding kit primers (Life Technologies, #4427046 and #4453189) and purified with Invitrogen PureLink^®^ PCR Micro Kit. Amplified DNA was quantified with Qubit^®^ dsDNA HS Assay Kit on a Qubit^®^ 2.0 fluorometer. Equal masses of each sample, containing specific barcodes, were pooled together and the mixture used as DNA template for emulsion PCR on Applied Biosystems SOLiD^®^ EZ Bead™ E80 system (#4453095). After 3′ end modification with terminal transferase, template beads were deposited onto glass slides and libraries sequenced on a SOLiD™ 4 system using multiplex fragment sequencing protocol that generates about 700 millions 50 bases short reads per run.

#### Data analysis

Illumina 2 × 100 paired-end RNA-seq reads were aligned to the *T. cruzi* Dm28c genome (GenBank accession number MBSY00000000) and human genome (version 19) using Bowtie2 v. 2.3.0 [35] in local mode, within three different settings: using default parameters, −-very-fast-local and --very-sensitive-local. Only reads mapped with an score higher than 180 were used for posterior analyses.

SOLiD 1 × 50 single-end reads were aligned using SHRiMP2 v. 2.2.3 [36] with the following arguments: --strata -h 80% --local --max-alignments 1000, in order to consider all top scoring multiple alignments of each read since the *T. cruzi* genome is highly repetitive [37, 38]; multiple alignments reads were further randomly attributed to a single aligned position. Only reads mapped with an score higher than 300 were used for posterior analyses.

For RNA-Seq gene coverage analysis, genes were divided in 100 equally proportional bins and the number of reads aligned to each bin were summed up from the SAM file [39] using custom Perl scripts; percentage of maximum coverage were calculated for each bin considering the higher count bin (for each sample) as 100%. In order to exclude redundancy in *T. cruzi* gene annotation and read counts, clusters of orthologous genes were determined by MCL algorithm v. 10-148, with an inflation value –I of 5.0 [40], based on nucleotide sequence similarity produced by BLAST+ v. 2.4.0 analysis and named hereafter Supra Genes (SG) (Additional file 1). SG read counts were obtained by summing the number of reads aligned to each coding sequence (CDS) of all members of SGs using the Perl programming language script. Low expressed Supra Genes were excluded from the analysis if counts per million (CPM) was smaller than one for at least two samples. Across sample SG count normalization was done by the TMM method [41] and differentially expressed Supra Genes detected by using the R v. 3.2.3 Bioconductor v. 3.5 package edgeR v. 3.12.0 [42], using a negative binomial model and an exact test based on quantile-adjusted conditional maximum likelihood (qCML) method. Alignment visualization was done in IGV v. 2.3.92 software [43]. Pearson correlations were calculated in R v. 3.3.1 using log_2_ of SG read counts.

To compare our transcriptome data to that from Li and collaborators [44], we took the raw read counts results from the SAM file (Table S6 from [44]) and correlated the CL Brener gene IDs to Dm28c Supra Gene IDs by BLAST+ v. 2.4.0 blastn search using the Esmeraldo haplotype data as query (Additional file 2), with default parameters except an e-value threshold of 1 × 10^−5^. The present results and Li and collaborators [44] data were processed together using our pipeline of RNA-Seq counts normalization, differential expression detection and correlation analysis as described above.

All RNA-Seq data have been deposited in NCBI’s Gene Expression Omnibus and are accessible through GEO Series accession number GSE94766.

## Results

### Establishment of T7SL IVT method and comparison to T7oligo(dT) IVT

**Fig. 1:**
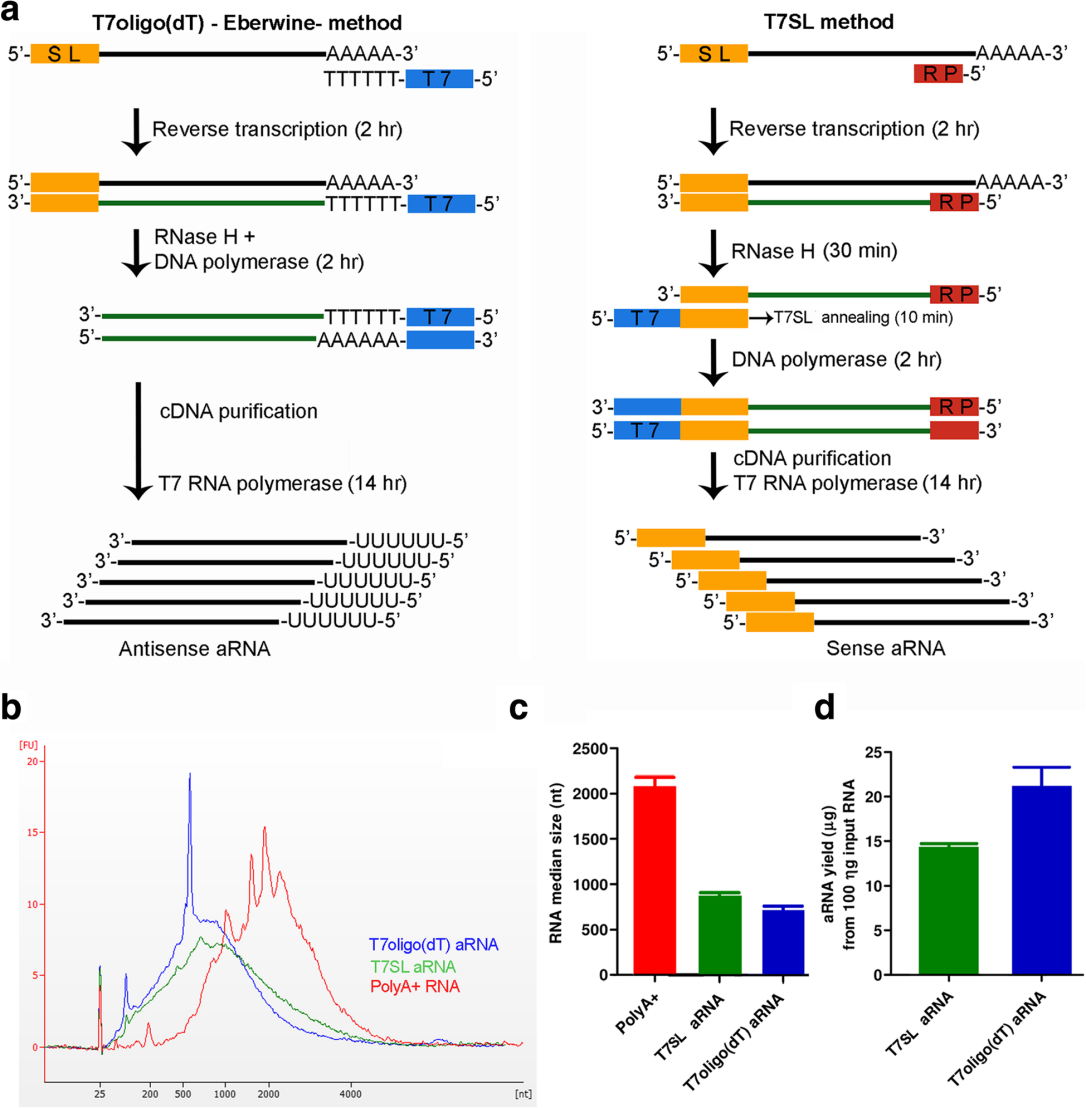
T7SL IVT method. **a** Comparison between T7oligo(dT) (left) and T7SL IVT (right) methods. In the Eberwine method, the reverse transcription reaction is performed using a T7oligo(dT) primer, resulting in a first-strand cDNA containing the T7 promoter. The T7 RNA polymerase is added for in vitro transcription of the purified cDNA and antisense aRNA is obtained. For the second method (T7SL IVT), the reverse transcription reaction is performed using random primers and the second-strand cDNA is obtained by a DNA polymerase reaction with the T7SL primer, which also contains the T7 promoter. After cDNA purification, in vitro transcription with the T7 RNA polymerase is performed, producing sense aRNA. **b** Distribution of RNA lengths from IVT samples and poly(A) + RNA (control), quantified by the BioAnalyzer 2100 equipment (Agilent). **c** Median length of poly(A) + RNA and aRNAs from the IVT samples. **d** aRNA yield obtained from the two IVT methods

To optimize the T7SL IVT method, we initially compared the amplified RNA (aRNA) profile with the aRNA generated by the classic Eberwine method that uses T7oligo(dT) primer. For specific amplification of mRNA bearing SL, we designed an oligonucleotide complementary to the last 21 bases at the 3′-end of *T. cruzi* spliced leader with an upstream T7 RNA polymerase promoter (see methods), which we named T7SL. First-strand cDNA was synthesized using random primers and, after RNA degradation, second-strand cDNA was produced using the T7SL primer (Fig. 1a). Distinct from T7oligo(dT) IVT procedure, where the T7 promoter is added to the first-strand cDNA, the T7SL IVT method attaches the T7 promoter to the second-strand cDNA (Fig. 1a). Hence, T7SL IVT will produce sense oriented aRNA, while T7oligo(dT) IVT produces anti-sense aRNA.

RNA amplified by T7SL IVT showed a smooth length distribution, consistent with a global amplification of all mRNAs of the parasite (Fig 1b). Although T7oligo(dT) showed a similar pattern, a RNA spike approximately at 600 nt was always present (Fig. 1b). When compared to purified poly(A) + RNA from *T. cruzi*, both amplification methods showed a smaller median RNA size (Fig. 1b and c). As discussed below, this is probably due to the 5′ and 3′ bias in T7SL and T7oligo(dT) amplification methods, respectively. The aRNA yield was similar for both methods (Fig. 1d); starting with 100 ng of total RNA, T7SL and T7oligo(dT) IVT generate an average of 14 and 21 μg of aRNA, respectively. Considering an empiric estimation that 3% of total parasite RNA correspond to mRNA (3 ng on a 100 ng total RNA sample), these yields are equivalent to 4718 and 7000-fold mass amplification of input mRNA, respectively.

### aRNA-Seq

**Fig. 2:**
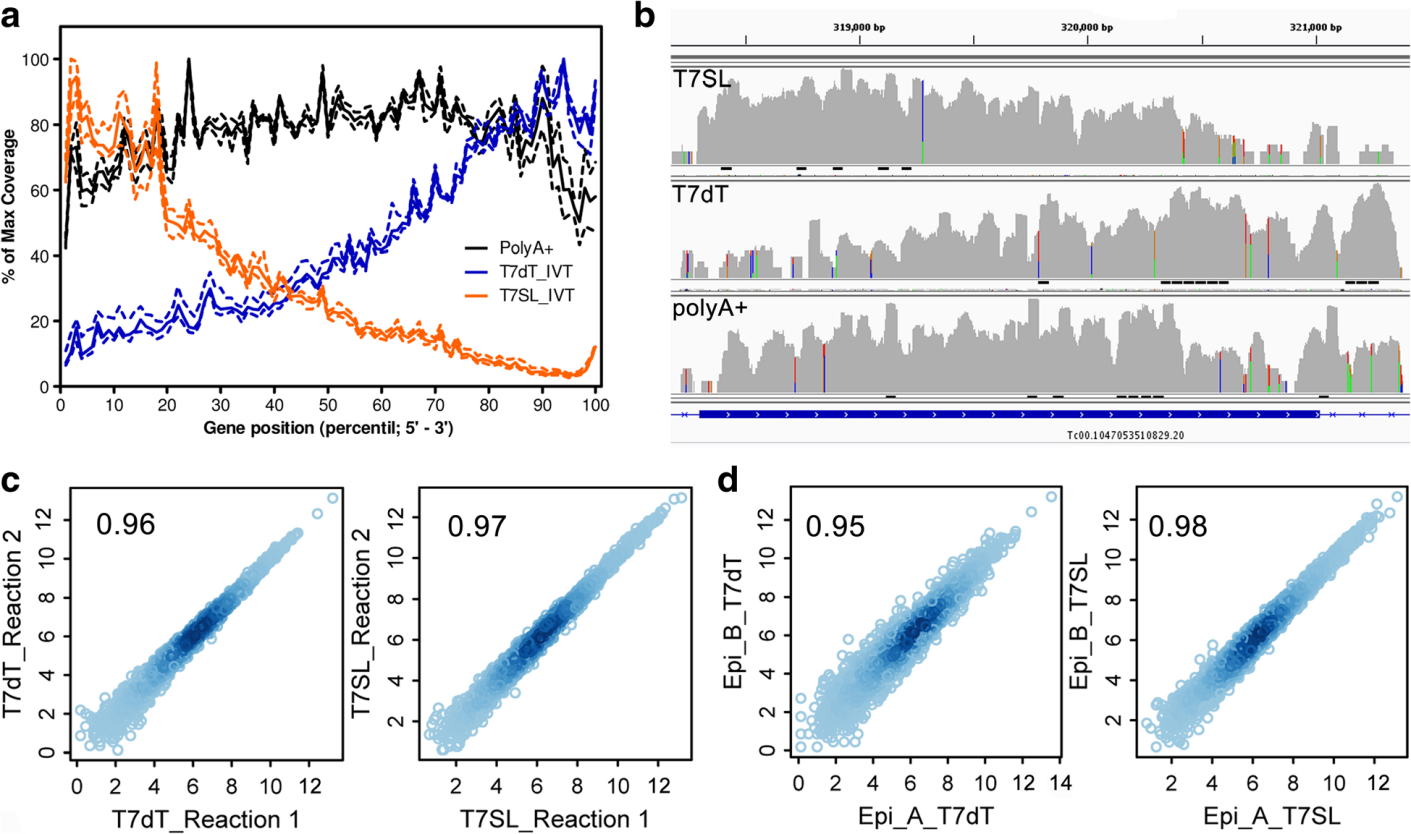
aRNA-Seq results. **a** RNA-Seq coverage along annotated genes. To plot all genes in the same graph, all coding sequences were split in 100 bins (percentiles) and the number of reads aligned to each percentile were summed and plotted as a ratio against the bin with higher number of aligned reads. Dotted lines are standard deviation. **b** IGV genome browser visualization of RNA-Seq reads alignment along a 3 kb gene for all three methods used (specified in left). Coverage were plotted in log scale. **c** correlation between the technical replicates (same RNA input for different amplification reactions) of T7SL IVT and T7oligo(dT) IVT methods. **d** correlation between biological replicates (RNA from separate epimastigote populations) of T7SL IVT and T7oligo(dT) IVT. For all scatter plots, scales are log_2_ of normalized read counts and values inside the graphs represent Pearson correlation

After initial optimization of T7SL IVT, we analyzed the aRNA composition by RNA-Seq, comparing our method to the usual T7oligodT IVT amplification and the gold standard RNA-Seq with purified poly(A) + RNA. We sequenced an average of 24 ± 4 million short reads (50 bases) for each one of the 44 samples in the SOLiD™ platform (Additional file 3). Visualization of aligned reads on the parasite genome showed that each RNA class (T7oligodT, T7SL, poly(A) + RNA) has distinct coverage profiles (Figs. 2a and b). While poly(A) + RNA presented a more even read distribution across annotated genes, as expected [5, 45], T7SL and T7dT have 5′ and 3′ bias, respectively, which is more evident for longer genes (Additional file 4: Figure S1). This is expected as each IVT method positioned the T7 promoter in different mRNA extremities (Fig. 1). Although each method has distinct gene coverage profiles, the detection of differentially expressed Supra Genes (DEGs) between epimastigotes and tissue culture-derived trypomastigotes is similar for the three methods (details below), as generally the biases are gene-specific. Raw and normalized SG read counts are shown in Additional file 5.

RNA-seq reads were aligned to our first version of *T. cruzi* Dm28c genome produced using Pacific Bioscience Technologies (PacBio) [46]. As this third generation sequencing technology generates reads in average longer than 8 kb, this draft version is of higher quality than the usual CL Brener reference genome, lowering the number of contigs from 32,746 to 1030, increasing the size of the larger contig from 0.26 MB to 1.54 MB and increasing contig N50 from 15 kb to 133 kb [37]. These improvements, as well as the fact that our mRNA samples were derived from Dm28, improve the alignment of RNA-Seq reads (68% of the reads aligned in a single position in Dm28c genome, instead of 56% in the CL Brener genome). There is another version of the Dm28c genome available at GenBank (AYLP00000000.1), but it has lower quality, as this version was sequenced using 454 technology and hence has a more fragmented contig distribution, with its larger contig having only 0.09 MB, besides the higher error rate in homopolymer tracts typical of pyrosequencing. This Whole Genome Shotgun project has been deposited at DDBJ/ENA/GenBank under the accession MBSY00000000. The version described in this paper is version MBSY01000000.

After aligning the RNA-Seq reads to *T. cruzi* genome, per Supra Gene read counts were computed. When comparing the technical replicates (same total RNA sample used as template for two independent amplification reactions), both IVT amplification methods have a very high correlation between technical replicates (Fig. 2c). After summing up read counts for IVT technical replicates, we also assessed the biological degree of reproducibility, using two independent biological samples for the same parasite stages, epimastigote and trypomastigote. For both IVT methods, biological variance is similar to the technical variance (Fig. 2d). Interestingly, the T7SL method presented a slightly higher average biological correlation in comparison to T7dT and poly(A) + RNA (Additional file 4: Table S1).

**Fig. 3:**
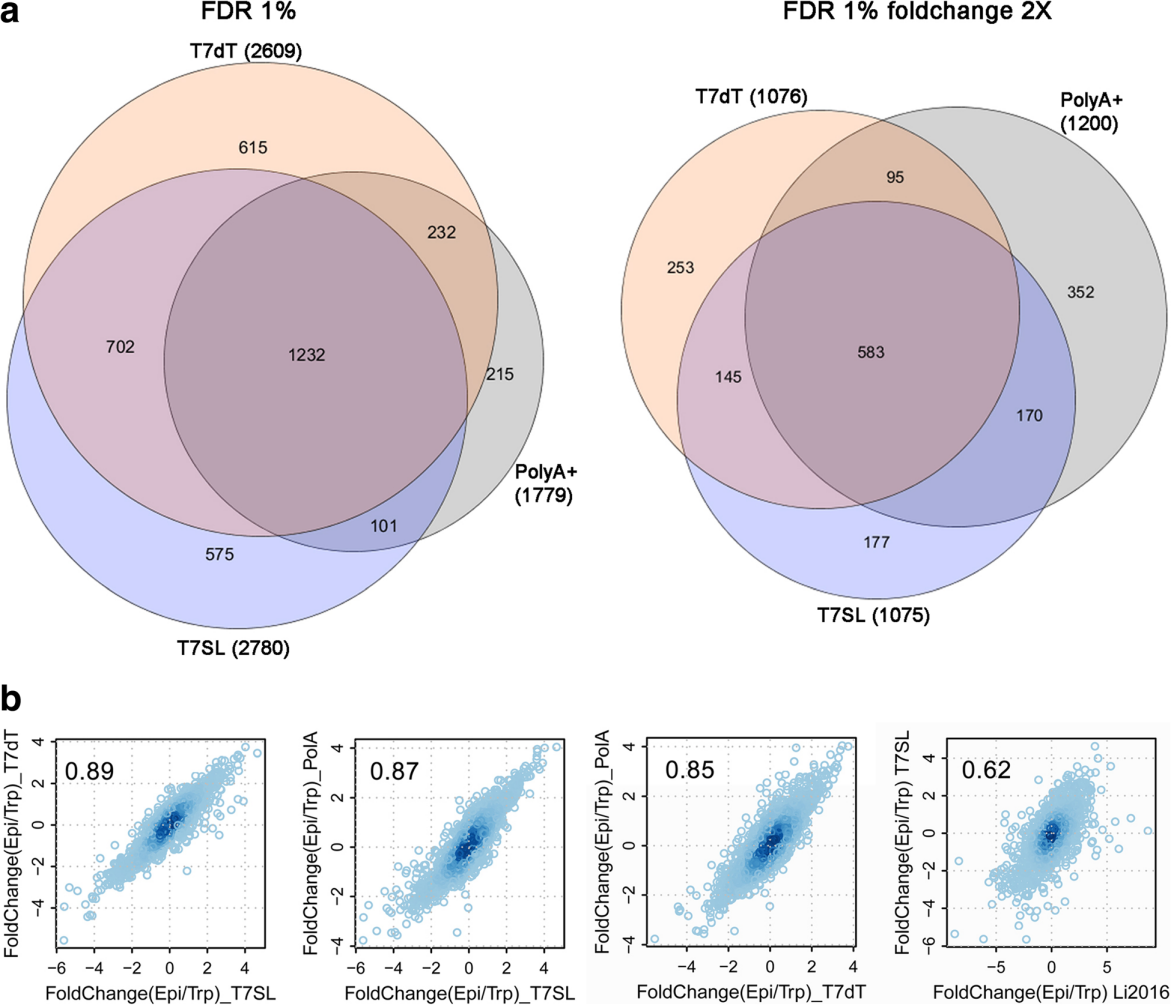
Putative DEG detection for the three analyzed methods. **a** Euler diagram showing the number and overlap of detected putative DEGs (FDR < 0.01) for each method (T7SL, T7dT and poly(A)+) when comparing epimastigote to trypomastigote transcriptomes, without a fold change threshold (left diagram) or at least a fold change of two (right diagram). Graphics produced on Cytoscape 3.2.0 **b** Scatter plot correlating epimastigote to trypomastigote fold changes (log_2_) for the different RNA-Seq methods; values inside the graphs represent Pearson correlation. Far right scatter plot correlates T7SL fold changes to the ones detected by our analysis of Li and collaborators data [44]

As RNA-Seq is typically used to detect DEGs, we applied all three methods (T7SL IVT, T7oligo(dT) IVT and poly(A) + RNA) to compare two different stages of *T. cruzi* life cycle, epimastigotes and trypomastigotes. At the same statistical significance level (FDR < 0.01), T7SL showed a higher number of DEGs, as this method detected 56.3% and 6.5% more putative DEGs in comparison to poly(A) + and T7dT methods (Fig. 3a). However, when using a twofold threshold in Supra Gene expression level, all methods showed a similar number of DEGs, indicating that those DEGs detected only on T7SL are of small fold change and probably due to an increased reproducibility. When co-visualizing the fold change, read counts and statistical significance of DEGs in a network representation (Additional file 4: Figure S2), it is evident that DEGs identified in all three methods are of higher fold change and/or read counts. T7SL method, however, has more capability of detecting low expressed or smaller fold change DEGs. This increased detection can be a technical bias, but generally it is considered that T7-amplification improves the mRNA quantification by decreasing variability, as mentioned above. A list of epimastigote to trypomastigote DEGs for each method used herein can be found in Additional file 6.

We computed the read count fold change (log_2_) for all parasite genes, and showed that epimastigote to trypomastigote fold changes are similar for all three methods (Fig. 3b). Higher correlation were obtained when comparing T7SL IVT to either one of the other methods, in comparison to T7dT versus poly(A) + RNA correlation, suggesting that T7SL is a reliable representation of fold changes distribution.

We compared the recently published *T. cruzi* transcriptome data [44] to present results. Normalized read counts showed low correlations when comparing same parasite stages (*r* = 0.26 to 0.56) (Additional file 4: Figure S3a), but this can be explained by several reasons, including (i) different parasite strains, (ii) distinct genome used for RNA-Seq alignments and (iii) distinct laboratorial procedures. However, when comparing the expression fold changes between epimastigotes to trypomastigotes (Fig. 3b and Additional file 4: Figure S3b), the correlation slightly improves (r~0.6), showing that, apart from distinct methods used, the biological meaning of both works was similar. When comparing epimastigote to trypomastigote DEGs (FDR < 0.01), about 64% (1463 of 2298) of significant genes detected using Li and collaborators data [44] were also detected in the present work (Additional file 4: Figure S4a). Furthermore, present T7SL data detected 952 epimastigote to trypomastigote DEGs that were not detected in Li data (Additional file 4: Figure S4a). When considering the most significative DEGs for T7SL method (FDR < 0.01, fold change > 2; 872 genes), about 53% (461) was also detected in Li data (Additional file 4: Figure S4b). Apart from the peculiarities of each method, 98% (448 of 461) of this common DEGs showed the same fold change direction (Additional file 4: Figure S4b-i and S4b-iv). Present data corroborate previous work on the *T. cruzi* steady state transcriptome [44, 47], showing the upregulation of several metabolism enzymes in epimastigote stage (Additional file 4: Figure S4b-i; Additional file 6) and several MASPs and trans-sialidases in trypomastigotes (Additional file 4: Figure S4b-iv; Additional file 6).

### T7SL IVT improves parasite transcriptome analysis in host-parasite RNA mixtures

**Fig. 4:**
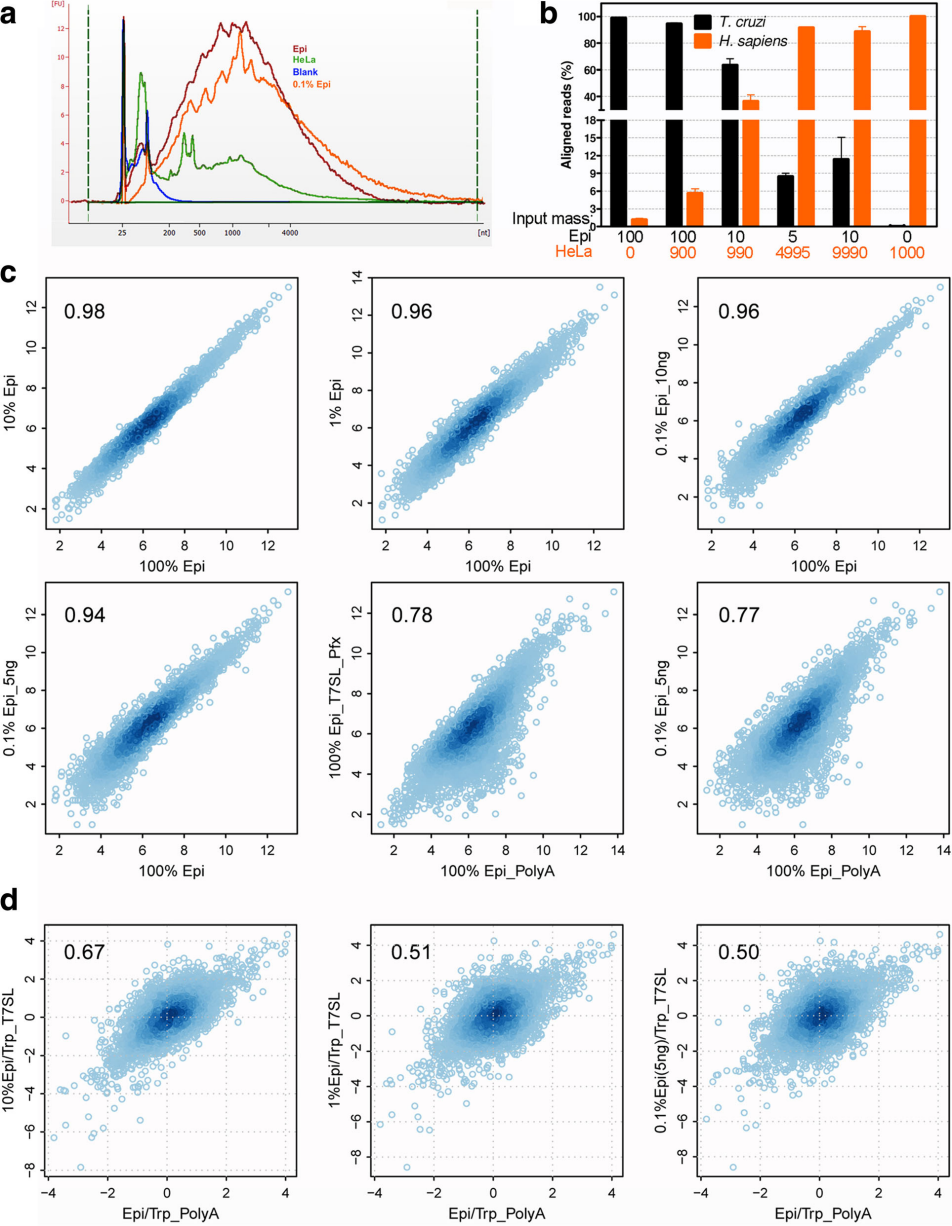
Performance of T7SL IVT on RNA mixtures. **a** Length distribution profiles for aRNA produced from pure samples (*T. cruzi* epimastigote and HeLa), mixture of *T. cruzi* (epimastigote) and HeLa and blank samples (no RNA for amplification reaction). Percentage are relative mass of Epi:HeLa on RNA mixture used for T7SL IVT. **b** After T7SL IVT and RNA-Seq of mixture samples, reads were aligned to *T. cruzi* and human genomes. The percentage of aligned reads with best match on parasite or human genome were retrieved and plotted. The input mass (in ng) used in each mixture is specified at the bottom. **c** Scatterplot comparing T7SL Pfx IVT and mixtures of HeLA-Epi RNAs, or PolyA+ RNA against HeLA-Epi RNAs. **d** Scatter plots of epimastigote to trypomastigote fold changes when comparing PolyA(+) transcriptome quantification to T7SL using *T.cruzi*/HeLa RNA mixtures

After the establishment and validation of T7SL IVT, we evaluated the specificity of parasite mRNA amplification by testing the reaction in mixtures of *T. cruzi* and HeLa RNA. The aRNA yield of T7SL IVT was around 43 times smaller when using HeLa RNA as input (340 ng aRNA produced from 100 ng input), but in order to increase specificity, we modified the protocol used for *T. cruzi* RNA samples, using Platinum^®^ Pfx DNA Polymerase for second-strand cDNA synthesis at 68 °C instead of 16 °C when using regular DNA polymerase. This modification did not affect the yield and pattern of *T. cruzi* RNA amplification (Fig. 4a) but decreases human aRNA yield in 20%.

We further tested the *T. cruzi* transcriptome quantification in the following *T. cruzi*/HeLa RNA mixtures: (i) 900 ng HeLa plus 100 ng Epi (10% parasite RNA), (ii) 990 ng HeLa plus 10 ng Epi (1% parasite RNA) and (iii) two mixtures containing 0.1% of parasite RNA (4995 ng HeLa plus 5 ng Epi and 9990 ng HeLa plus 10 ng Epi). The 0.1% mixtures corresponds to about 10^6^ human cells (around 5 μg of total RNA) together with 10^4^ epimastigotes (around 5 ng of total RNA, see Additional file 4: Table S2). Bioanalyzer analysis demonstrates that the 0.1% parasite RNA mixture generate aRNA with length distribution profile resembling the one from *T. cruzi* aRNA, but with a less smooth pattern (Fig. 4a).

We conducted RNA-Seq experiments on aRNA generated from mixture samples and the resulting reads were aligned to a *T. cruzi*-*H. sapiens* concatenated genome in order to observe the percentage of aligned reads with best match on the parasite or human genome. For the 10% parasite RNA sample, around 94% of aligned reads were best aligned to *T. cruzi* genome (Fig. 4b); for the 1% parasite RNA sample, more than 60% of aligned reads showed a best match to the parasite genome; and when decreasing the parasite RNA proportion to only 0.1% of total mass, around 10% of the aligned reads have a best match to the parasite genome. Although this means a lower sequencing coverage for samples with low proportion of parasite mRNA, it is a significant reduction in read waste when comparing to mixture RNA samples without amplification, as 10% of aligned reads on a 0.1% original sample represents a 100-fold enrichment for parasite RNA. For HeLa pure samples, only a very small proportion of aligned reads (~0.02%) showed a better alignment to the *T. cruzi* genome (detailed below).

When comparing read counts per Supra Gene for pure and mixed samples using T7SL Pfx IVT aRNA (Fig. 4c), the parasite transcriptome quantification was very reproducible with Pearson correlations above 0.94 even for the 0.1% parasite RNA samples (Fig. 4c). Finally, we visualized read counts scatter plots when comparing epimastigote poly(A) + RNA to epimastigote T7SL Pfx IVT aRNA generated in the most diluted samples (Fig. 4c). When using the optimized protocol for T7SL Pfx IVT from 100 ng of pure parasite RNA, Pearson correlation was 0.78 to Poly(A) + RNA. Interestingly, aRNA generated from host-parasite RNA mixture containing only 0.1% of parasite RNA had a similar Pearson correlation (*r* = 0.77). This result indicates that T7SL amplification method performs better, in general, than PCR based mRNA amplification, since a recent study in *T. brucei* based on this technique reported a correlation of 0.38 [31] between poly(A) + RNA and SL-based PCR amplified RNAs. Scatter plots of epimastigote to trypomastigote fold changes showed that when the percentage of epimastigote RNA on the mixture diminishes, the correlation to poly(A) + transcriptome decreases from 0.67 (10% epimastigote mass) to 0.50 (0.1% epimastigote) indicating a higher impact of stochastic factors when the parasite RNA input is too low (Fig. 4d).

**Fig. 5:**
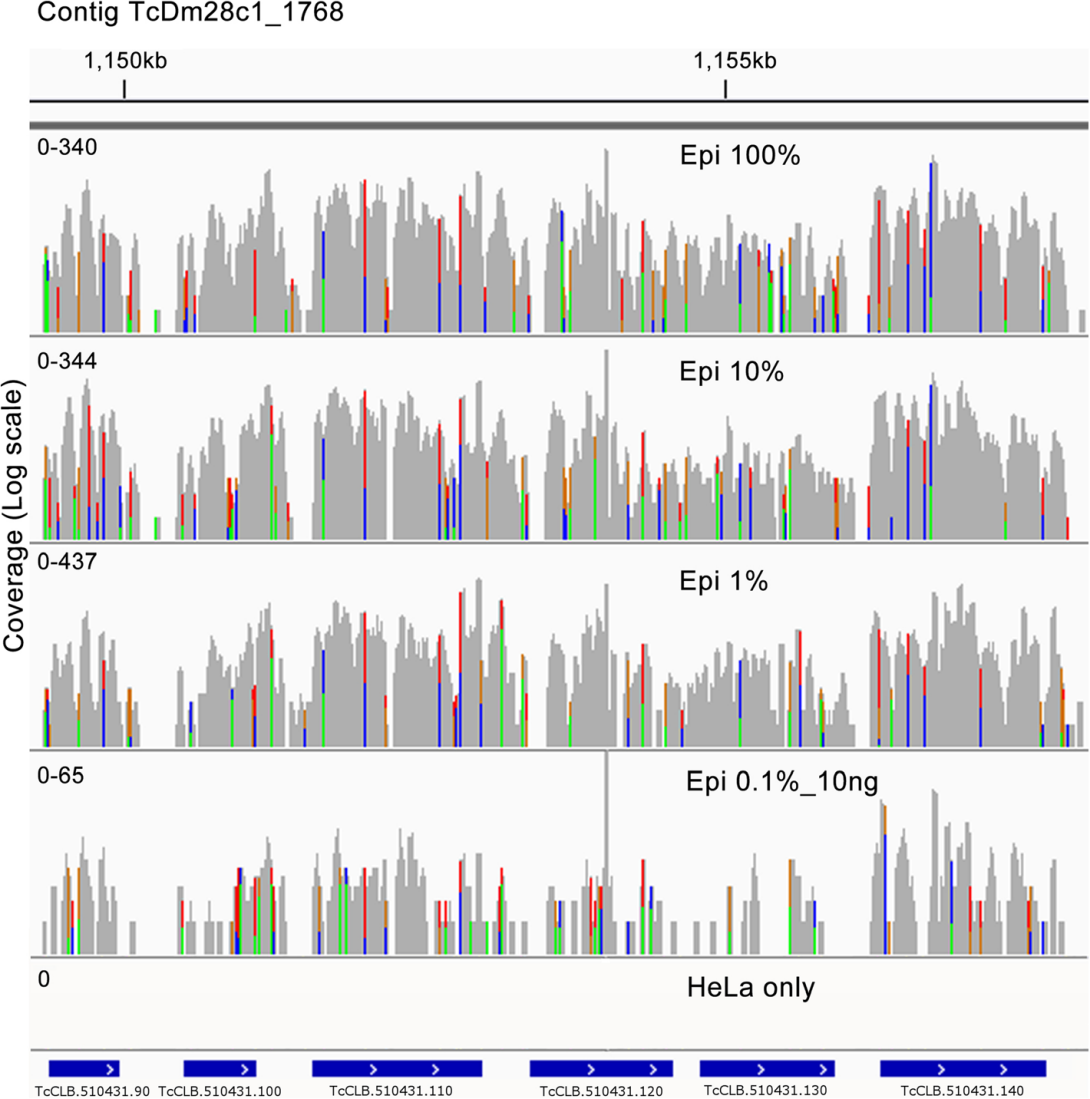
IGV view of RNA-Seq aligned reads coverage for *T.cruzi*-HeLa mixture samples. A ~8 kb genome region, containing six genes (blue boxes at the bottom), is showed. For each sample, y axis is log10 of coverage, with counts range specified at the left. Percentage of epimastigote RNA in the *T. cruzi*-HeLa RNA mixture used for T7SL Pfx amplification is indicated for each sample

Visualization of RNA-Seq reads alignment on parasite genome showed that even for *T. cruzi*-HeLa mixtures containing only 0.1% of epimastigote RNA, T7SL amplification generate a similar coverage throughout the genome (Fig. 5). It is worth to mention that T7SL aRNA-Seq of samples generated from HeLa pure RNA illustrates that HeLa amplified material has no significant impact in distorting *T. cruzi* read count per CDS. Apart the extremely low level of reads mapping to the *T. cruzi* genome (~0.02%), the great majority of them (~80%) are mapped in or nearby a 186 nt repetitive element that are close to the rDNA gene cluster (Additional file 4: Figure S5). Interestingly, there is no sequence similar to this repetitive element in the reference human genome. One possible explanation is that a similar sequence is present in the HeLa genome, but not in the reference human genome.

### Minimal RNA mass input for optimal T7SL IVT

**Fig. 6:**
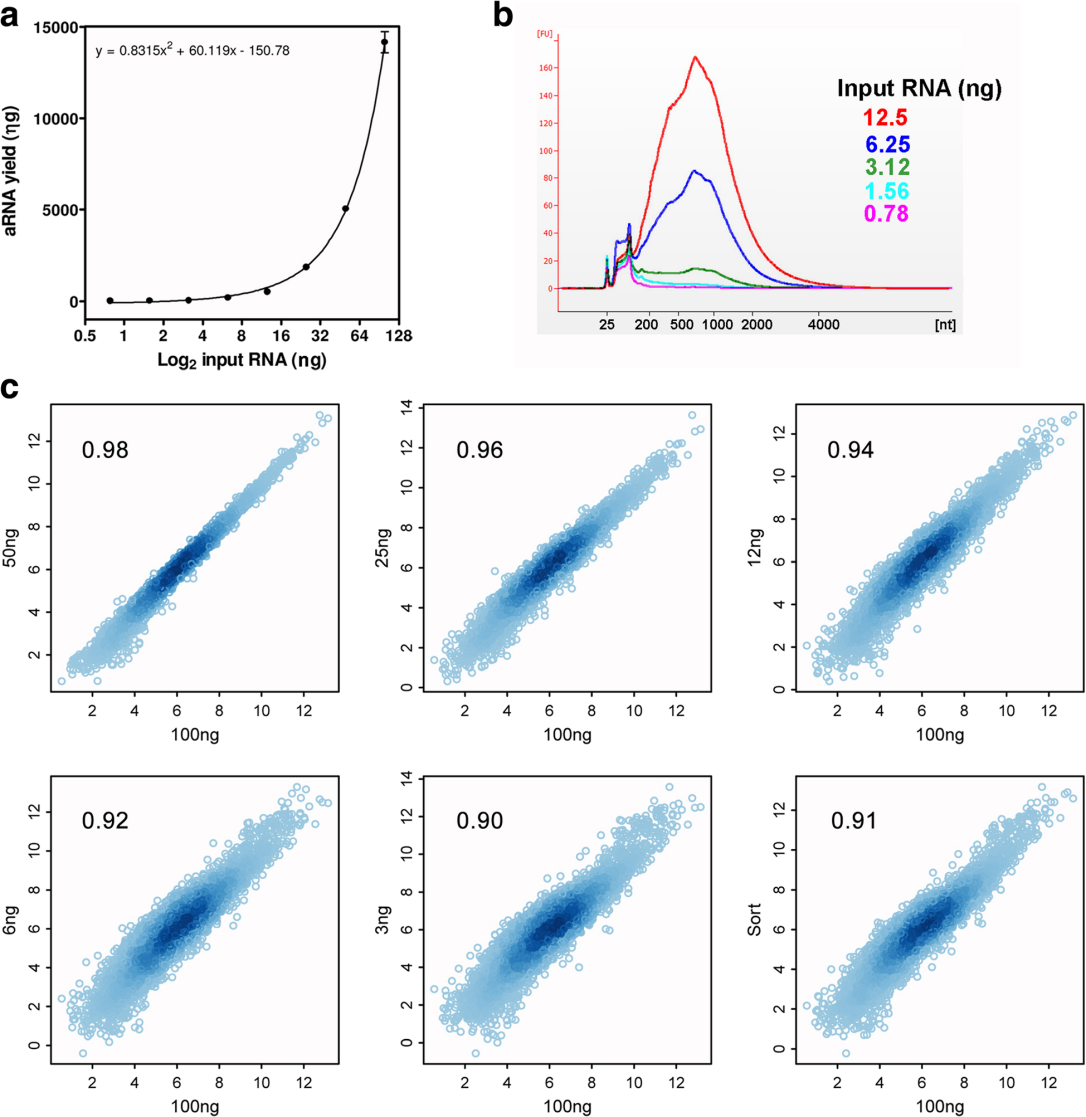
Minimal input RNA mass for optimal T7SL IVT. **a** Graph showing the correlation between the input total RNA mass (X axis) and the aRNA yield (Y axis). **b** aRNA length distribution obtained with different input RNA mass. Note that below 6.25 ng input, the aRNA lose the typical smooth length distribution. For all samples, the same RNA mass were applied on a BioAnalyzer chip. **c** RNA-Seq scatterplot with different RNA inputs (from 100 to 3 ng) for T7SL IVT. Pearson correlation is depicted, based on log normalized read counts. Note that even when 3 ng of mass input is used for RNA amplification, the transcriptome quantification is very similar across all expression levels. “Sort” sample correspond to 10^5^ epimastigotes sorted directly to RNA extraction buffer, which roughly corresponds to 68 ng of total RNA (Additional file 4, Table S2)

Finally, we evaluated the minimal mass of input RNA allowing efficient mRNA amplification without significant bias. Serial twofold dilutions from 100 to 0.78 ng were used and the corresponding aRNA yield was non linearly related to input total RNA mass, since these variables follow a second order polynomial function (Fig. 6a). From the aRNA yield function, we estimated that 4 ng of input total RNA allows the generation of 100 ng of aRNA. aRNA length distribution have a similar smooth pattern down to 6 ng of input RNA; smaller inputs produce artifact small aRNA molecules of about 100 nt (Fig. 6b). This artifact small aRNA also appears when the T7SL amplification yield was smaller (using HeLa pure RNA) or mainly when using no RNA for amplification (blank samples, Fig. 4a).

We sequenced T7SL IVT aRNA generated from 100 to 3 ng of input total RNA and also from 100,000 sorted epimastigotes (around 68 ng of input total RNA). Results showed that even when pushing down the input mass to 3 ng, the transcriptome quantification shows a very good correlation (*r* = 0.90) to Poly(A) + RNA. In general, the global RNA quantification distribution is maintained, hence even with few nanograms of RNA (or 100,000 sorted parasites) we can quantify the parasite transcriptome with satisfactory precision (Pearson correlation higher than 0.9, Fig. 6c).

### T7SL enable DEGs detection independently of parasite RNA mass input and purity

**Fig. 7:**
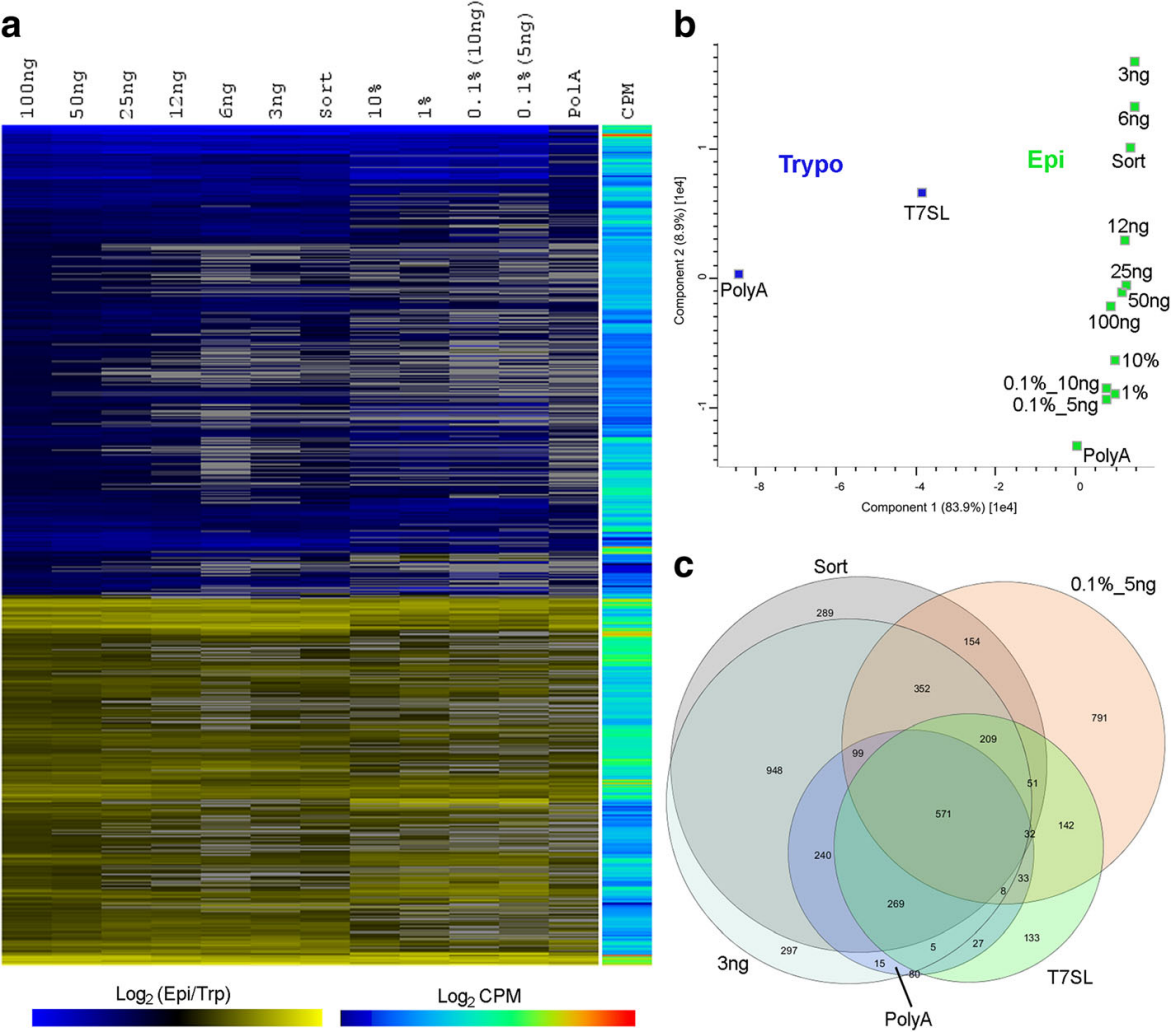
T7SL IVT DEGs detection on mixtures and limiting mass samples. **a** Hierarchical clustering of all DEGs (FDR < 0.01) when comparing all epimastigotes samples (mixtures and limiting mass) to the trypomastigote stage. Blue/black/yellow color code is log_2_ of expression fold change of epimastigote to trypomastigote). Supra Gene expression level is rainbow color coded based on log_2_ of counts per million reads (log_2_ CPM). Heatmap was created using Euclidian distance, average linkage clustering method and MeV (Multiple Experiment Viewer) v. 4.8.1 software. Note that, in general, all epimastigote samples show a similar fold change pattern for the great majority of genes, independent of initial mass used for amplification (limiting mass samples) or percentage of parasite RNA on host-parasite mixtures (mixture samples). **b** Principal Component Analysis (PCA) of the same samples used in **a**. The first component represents 84% of the total variation and is mainly due to epimastigote to trypomastigote differences; the second component represents 9% of the total variation and is mainly due to epimastigote transcriptome differences between the limiting mass experiments. PCA graph was created using Perseus v.1.5.0.31 software. **c** Euler diagram showing the number and overlap of detected DEGs for epimastigote to trypomastigote comparison using five different groups: (i) Poly(A)+: both parasite stages analyzed by Poly(A) + RNA; (ii) T7SL: both parasite stages analyzed by T7SL IVT amplification method from 100 ng of initial mass; (iii) Epi_3ng: limiting mass of 3 ng for epimastigote T7SL IVT; (iv) Epi_0.1%: mixture of host-parasite RNA containing only 0.1% of parasite RNA; (v) Epi_Sort: amplification of RNA obtained from 10^5^ sorted epimastigotes

To observe if the limiting percentage of parasite RNA on mixture samples influenced the quantification of DEGs, we compared all epimastigote experimental points (from 100% to 0.1% of relative parasite mass) to the trypomastigote stage, as a reference sample. Besides, we included the limiting mass samples on the same analysis. After detecting DEGs in all comparisons to trypomastigote, we plot in a heat map the 1079 most confident ones accordingly the optimal T7SL condition: epimastigote (100 ng, T7SL) versus trypomastigote (T7SL)(FDR 0.01 and twofold change). Interestingly, Fig. 7a shows that almost all experimental points (mixtures or limiting mass) have the same expression change direction when comparing epimastigote to trypomastigote. Those few Supra Genes that were detected with FDR < 0.01 and have discrepant fold change directions between samples are low read counts Supra Genes (9 of 1079 genes), which are naturally more prone to quantification stochastic variations (Fig. 7a). Principal component analysis showed that complex mixture samples or limiting mass of parasite RNA have a similar total transcriptome quantification to pure epimastigote samples, being that each group of samples (mixtures or limiting mass) clustered in PCA plot distinctly separated from the trypomastigote samples (Fig. 7b). This is not surprising given that the epimastigote and trypomastigote stages have very distinct mRNA profiles and that the T7SL IVT method displays low technical bias. It is interesting to notice that although mixture and limiting mass samples are closer to each other, they cluster in two distinct groups.

Next, we compared the identified DEGs in trypomastigote versus epimastigote samples, using five distinct comparisons: (i) purified poly(A) + RNA obtained for both parasite stages (gold standard RNA-Seq method), (ii) pure epimastigote aRNA produced from 100 ng of total RNA, (iii) the most limiting mass used for T7SL IVT (3 ng of epimastigote RNA), (iv) the lower percentage of epimastigote RNA in a host-parasite mixture (0.1% of parasite RNA) and (v) epimastigote RNA obtained by sorting of 10^5^ parasites. Comparisons (ii) to (v) were against T7SL IVT trypomastigote aRNA. After independent calculation of DEGs for each comparison, we compare the list of DEGs (considering an FDR of 1%) and plotted the overlap of DEGs in an Euler diagram (Fig. 7c). A vast majority of putative DEGs detected by gold standard poly(A) + RNA method were also detected by T7SL IVT method, even when using a limiting input mass of 3 ng. In general, T7SL IVT has reliable performance in DEGs detection even in the most extreme conditions of low input or low percentage of parasite RNA.

## Discussion

Direct RNA-Seq analysis of parasites in their hosts is a difficult task due to the low amount of RNA available to be extracted directly from infected tissue in comparison to the higher amount of co-purified host RNA. When the parasitemia is relatively high, typical poly(A) + RNA-Seq can be used to simultaneously capture the parasite and host cell transcriptomes, as recently showed for *T. cruzi* [44, 48], *T. brucei* [49] and Leishmania [50]. Li and collaborators [44] analyzed the *T. cruzi* transcriptomic modulation associated with the establishment of intracellular *T. cruzi* infection. In the initial times of infection (4 to 24 h), when the number of parasites to host cells is not higher than 1:1, generally less than 5% of the mixed host-parasite read pool mapped to the *T. cruzi* genome, reflecting the larger proportion of the host cell transcriptome. Hence, in cases where the relative number of parasites to host cells is much smaller than 1:1, the fraction of reads mapping to parasite genome will be too small. In this scenario, the parasite specific mRNA amplification method showed here is of great utility. The *T. cruzi*/HeLa mixed samples showed that it is possible to quantify the parasite transcriptome even when only 0.1% of total RNA mass correspond to parasite RNA, which is similar to a mixture of 10^6^ human cells and 10^4^ *T. cruzi* epimastigotes. In this case, typical poly(A) + RNA-Seq will not efficiently capture the parasite transcriptome and the present method is especially useful for low parasitemia samples or low input parasite total RNA. Considering that all mRNAs from trypanosomatids possess the same specific sequence at the 5′ region, the spliced leader or mini-exon, they could be selectively amplified through a SL-bearing primer. Although the SL trapping method theoretically provides a means of analyzing parasite transcriptome in complex RNA samples (such as host-parasite mixtures) [24], this hypothesis was only recently addressed by Mulindwa and collaborators [31]. Using a method similar to SL trapping, these authors synthesize *T. brucei*-specific cDNA by priming with a SL-specific oligo, followed by PCR amplification using nested primers to evaluate the *T. brucei* transcriptome in host-parasite RNA mixtures consisting of only 0.1% of *T. brucei* RNA. Their PCR based amplification of SL-containing mRNAs allowed comparison of different samples as long as they were all treated in the same way, but introduced significant bias when comparing to gold standard poly(A) + RNA-Seq [31].

Our method uses a different procedure, IVT for RNA amplification, as it linearly amplifies original mRNAs [8]. As already pointed out by Mulindwa and collaborators [31], SL priming followed by PCR-based cDNA amplification distorted relative abundances of the cDNA products and cannot be used, by itself, to measure absolute mRNA levels. Although the present T7SL IVT method also showed some bias on transcriptome quantification, the amplification distortion was smaller. When comparing transcriptome read counts distributions, the T7SL IVT method showed higher correlations to poly(A) + RNA (*r* = 0.77, for the 0.1% mixture) in comparison to PCR-based method (*r* = 0.34) [31]. This improvement is important to increase the ability to detect true changes in the expression profile, specially with low amount RNA or low ratio of parasite to total RNA samples. Furthermore, if comparing samples produced by the same T7SL IVT protocol, the correlation was very good even when using complex mixed samples or low input RNA, indicating that the bias created by the method, when compared to poly(A) + RNA, are gene-specific and not influenced by the RNA sample complexity.

The higher ability to detect epimastigote versus trypomastigote DEGs when using samples produced by the T7SL IVT method corroborate the idea that linear RNA amplification enables a more reproducible transcriptome quantification [12, 13, 17]. When analyzing samples with very low amount of parasite RNA, the lower proportion of parasite reads can be resolved by a larger amount of sequencing data, obviously with significant increase in cost. Even though T7SL IVT amplifies host RNA, present method showed at least 100-fold enrichment of parasite mRNA on resulting RNA-Seq reads that can be directly translated to a similar decrease in sequencing costs.

Other useful characteristic of the T7SL IVT method is the 5′ bias of the generated reads. Due to the fact that 5′-UTR regions are significantly smaller than 3′-UTR [51], usually 30 to 70 nt, a higher proportion of reads will align inside CDS regions. These regions are more diverse than UTRs and hence their correct mappability is higher in general.

## Conclusions

We concluded that the T7SL IVT method has several advantages for those researchers preparing trypanosomatid RNA-Seq libraries whenever the parasite RNA mass is a concern. As long as all samples are treated equally, T7SL IVT allows a powerful detection of putative DEGs in complex samples, including low input and host-parasite mixtures (especially for low parasitemia samples).

Although we developed T7SL IVT method using *T. cruzi* total RNA as template for amplification, this method can be easily adapted for any trypanosomatid species by only modifying the T7SL oligonucleotide. T7SL IVT opens new perspectives for trypanosomatid studies, specially parasite transcriptome analysis directly in their hosts.

## Additional files

Additional file 1: Shows the description of each *T. cruzi* cluster of orthologous genes, named as Supra Genes (SG) in this article. The CDS nomenclature (TcCLB) is from *T. cruzi* CL Brener genome annotation. (XLSX 278 kb)

Additional file 2: Shows the correspondence of each Dm28c Supra Gene to CL Brener Esmeraldo haplotype ID here used to compare the present work with that of Li and collaborators [44]. (XLSX 589 kb)

Additional file 3: Summary of all samples analyzed in present work containing results of RNA-Seq alignments and SRA accession IDs. (XLS 24 kb)

Additional file 4: Contains all supplemental figures (Figures S1 to S5) and tables (Table S1 and S2). (DOCX 1811 kb)

Additional file 5: Contains raw and normalized SG reads counts for all RNA-Seq samples. Detailed descriptions of all sample names are shown in Additional file 3. (XLSX 6607 kb)

Additional file 6: Contains lists of epimastigote to trypomastigote DEGs for each method used in the present article (PolyA+ RNA, T7SL and T7dT). (XLSX 1545 kb)

## Abbreviation

aRNA: Amplified RNA
CDS: Coding DNA sequence
DEGs: Differentially expressed genes
Epi: *T. cruzi* epimastigotes
FDR: False discovery rate
IVT: in vitro transcription
LogCPM: log_2_ of counts per million reads
PCA: Principal component analysis
SG: Supra Genes (cluster of orthologous genes in *T. cruzi* genome)
SL: Spliced leader
T7dT: Oligonucleotide containing a T7 RNA pol promoter and a downstream poly-T sequence
T7SL: Oligonucleotide complementary to the last 21 bases of *T. cruzi* SL sequence, bearing an upstream T7 RNA pol promoter
Trp: *T.cruzi* trypomastigotes
