# Supplementary material for: Trypanosoma cruzi specific mRNA amplification by in vitro transcription improves parasite transcriptomics in host-parasite RNA mixtures
Source: BMC Genomics. 2017 Oct 16;18:793. doi: 10.1186/s12864-017-4163-y (PMC5644099; doi:10.1186/s12864-017-4163-y)
Supplement: Supplementary file 4 — Contains all supplemental figures (Figures S1 to S5) and tables (Table S1 and S2). (DOCX 1811 kb) [file 12864_2017_4163_MOESM4_ESM.docx]

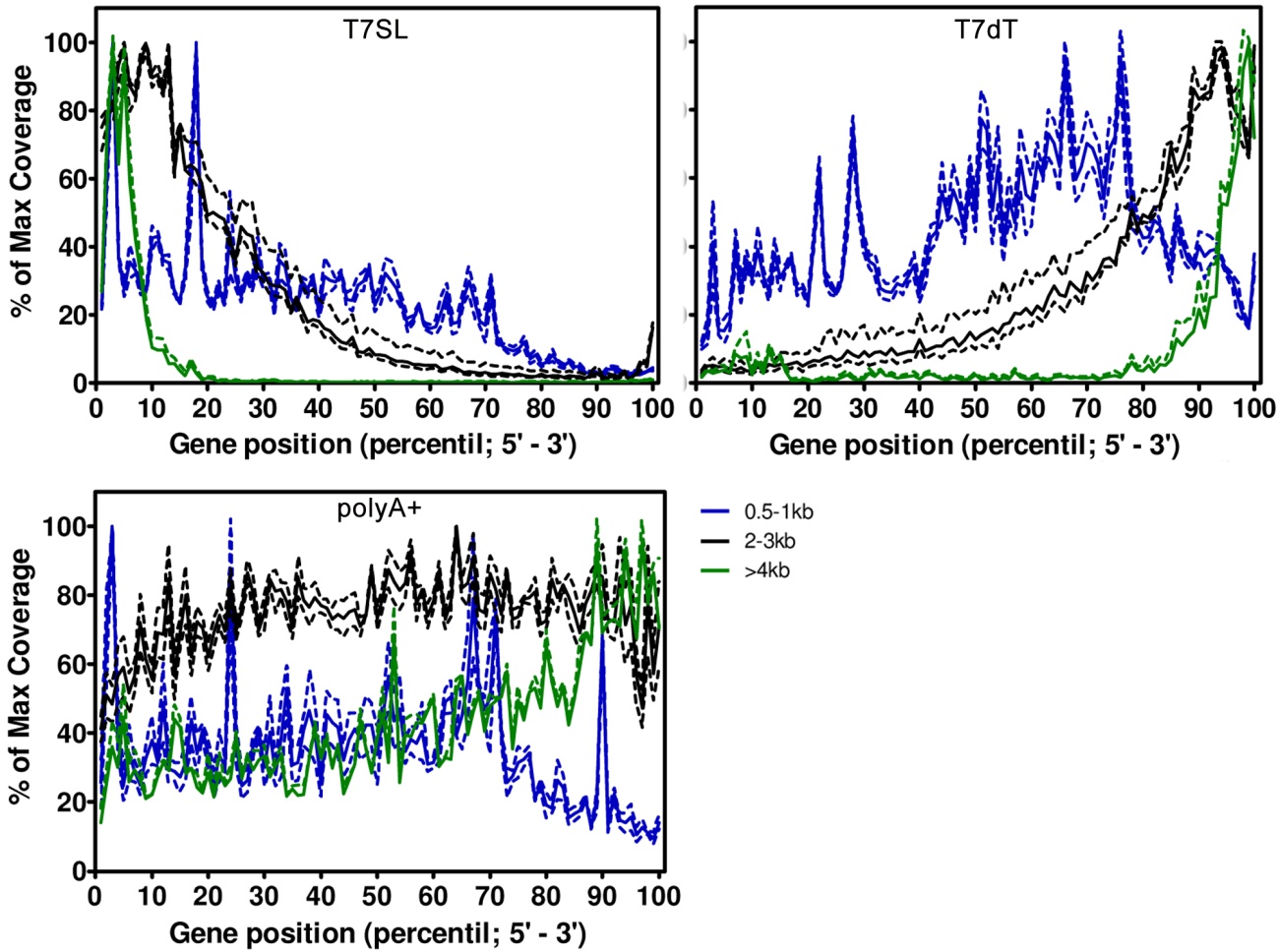


**Fig S1. RNA-Seq coverage in relation to gene size.**

Annotated parasite genes were split in three groups based in length (0.5-1 kb, 2-3 kb and >4 kb). Plots were constructed in the same way as in Figure 2A.

**
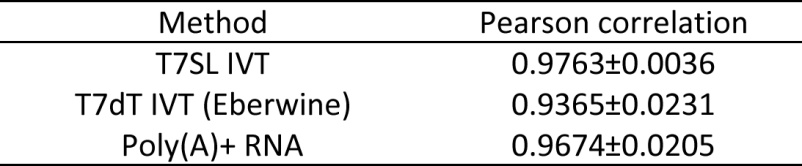
**

**Table S1. Biological correlation between RNA-Seq read counts.**

Pearson correlation for read counts distributions were obtained after normalization and log_2_ transformation of raw counts. Values correspond to average and standard deviation.


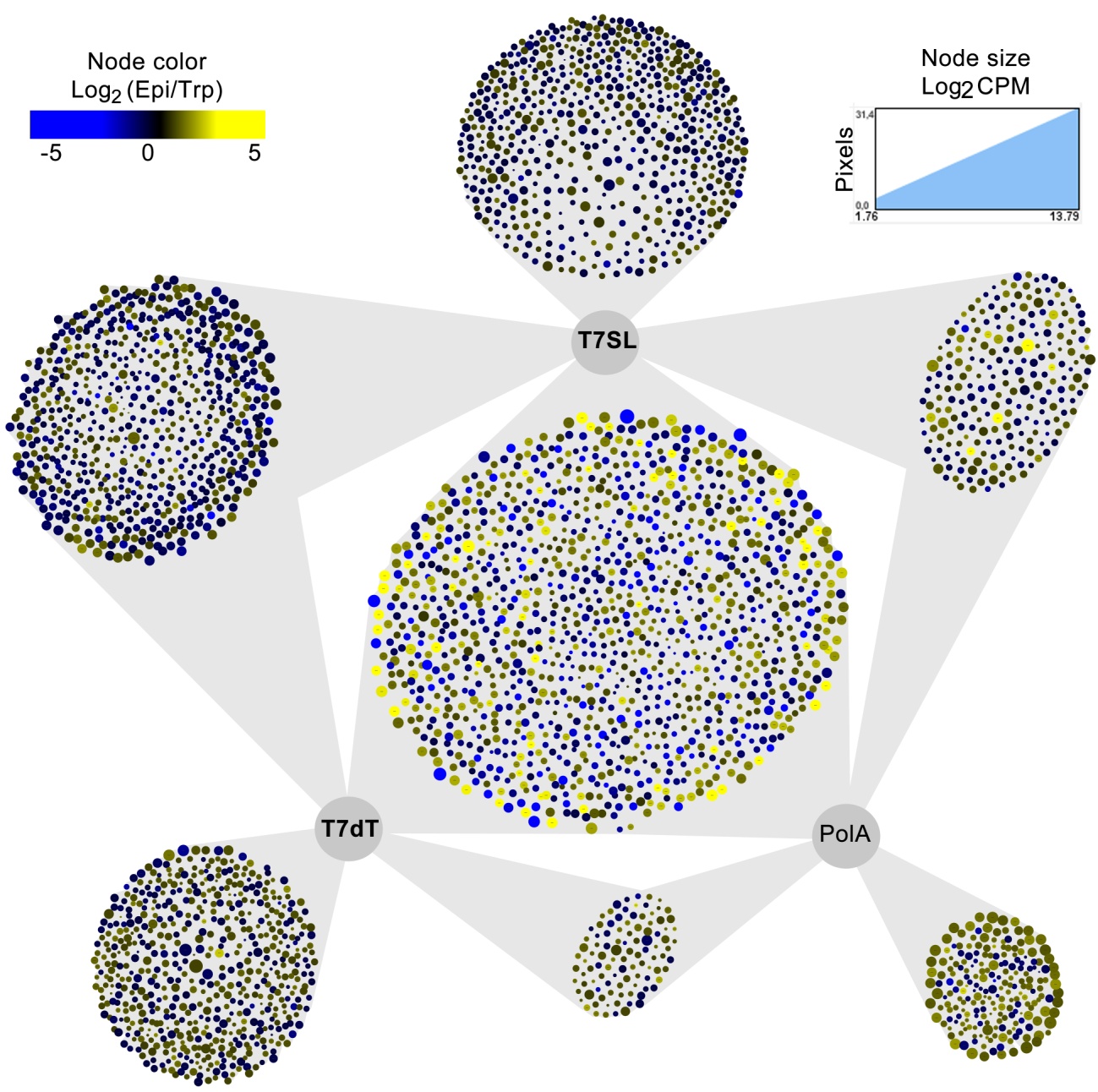


**Fig S2. Network representation of shared DEGs**

Network representation of shared DEGs between each method together with fold change scale and read counts (denoted by node size, depicted in log2 counts per million reads). Network was constructed using the Cytoscape software.


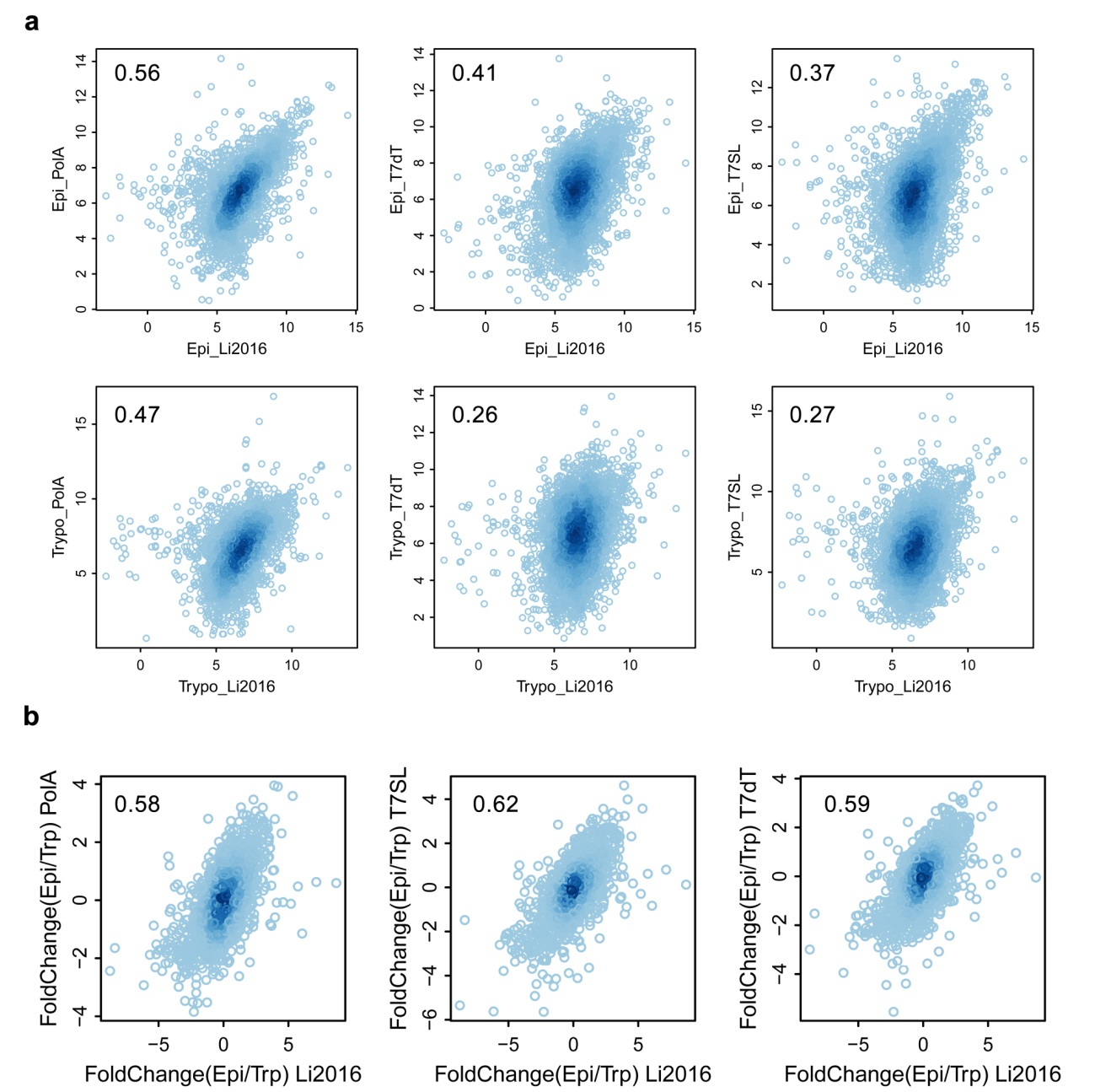


**Fig S3. Comparison of RNA-Seq data from present work to that from Li and collaborators (2016)**

**a** Scatter plots showing the correlative expression level (log_2_ normalized cpm) between different samples of present work and from Li et al 2016. **b** Scatter plots of fold changes between epimastigote to trypomastigote expression levels. Values inside the graphs represent Pearson correlation.


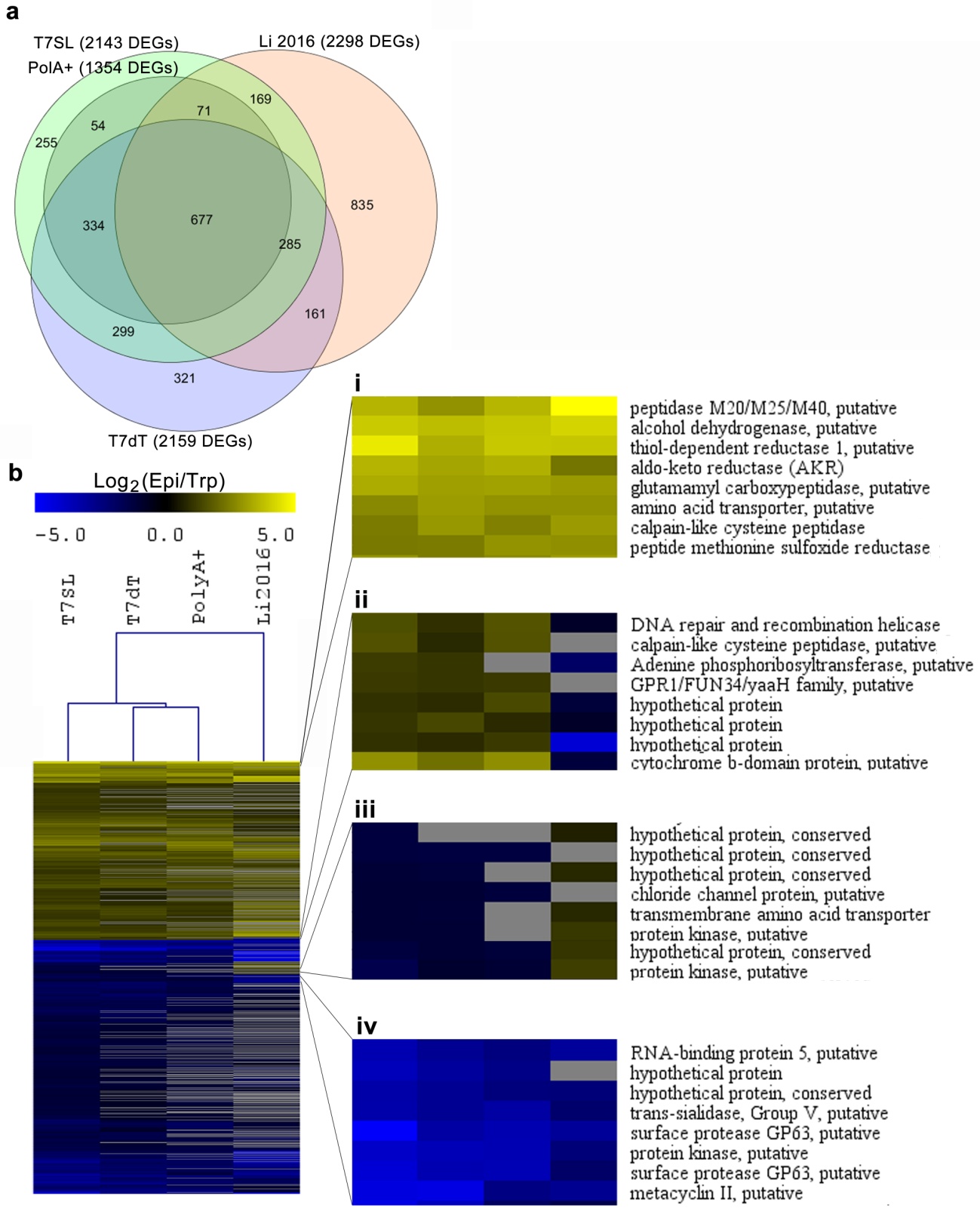


**Fig S4. Comparison of differentially expressed genes detected in present work and in Li *et al* (2016)**

**a** Euler diagram showing the overlap of DEGs for each method. **b** Heat map of epimastigote to trypomastigote fold-change for the compared methods. In general, all methods showed the same fold change direction for all DEGs. Some regions of the heat map are highlighted for clarity. Details in main text.


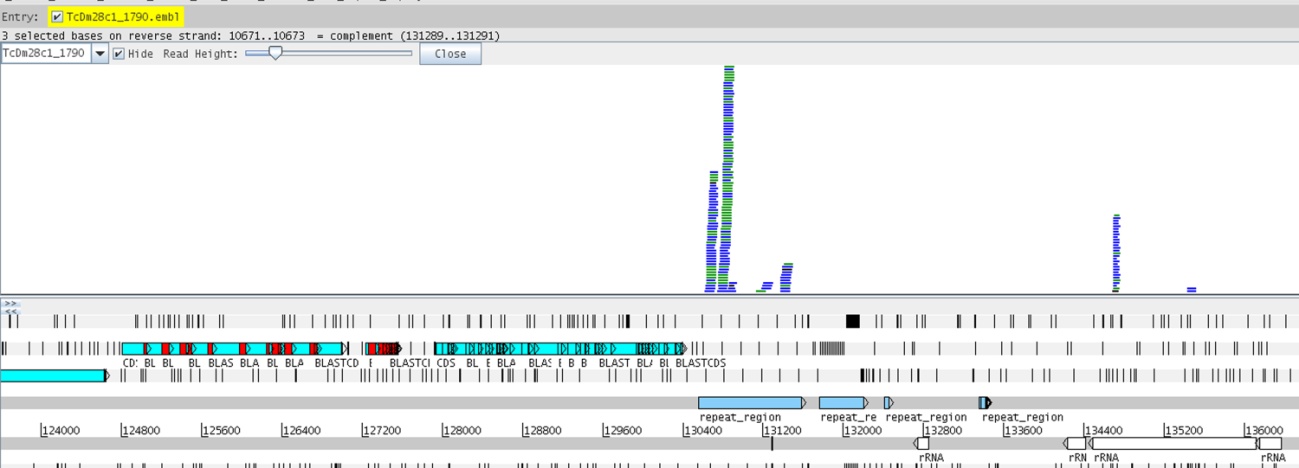


**Fig S5. Mapping of HeLa T7SL aRNA-Seq reads to the *T. cruzi* genome**

Artemis Genome Browser visualization of alignments. Apart the extremely low level of HeLa aRNA reads mapping to the *T. cruzi* genome (~0.02%), the great majority of them (~80%) are mapped in or nearby a 186 nt repetitive element that are close to the rDNA gene cluster. A region of ~13 kb in the largest genome contig (TcDm28c1_1790) is highlighted to show the reads alignment.


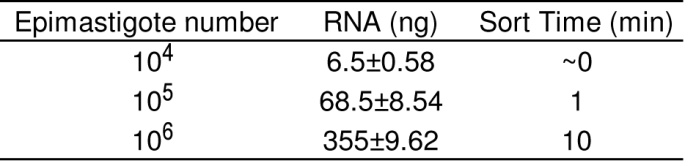


**Table S2. Total RNA mass recovered from sorted epimastigotes.**

The indicated number of T. cruzi epimastigotes were sorted directly to RNA extraction buffer (RLT buffer from RNeasy kit - Qiagen^TM^), using a BD FACS AriaII cell sorter. RNA was purified and quantified in the same way as described in manuscript methods. The indicated sort time was obtained for an epimastigote pure culture (sort gate on ~80% of total events).
